# Public Health Aspects of Climate Change Adaptation in Three Cities: A Qualitative Study

**DOI:** 10.3390/ijerph191610292

**Published:** 2022-08-18

**Authors:** Gloria Macassa, Ana Isabel Ribeiro, Anneli Marttila, Frida Stål, José Pedro Silva, Michelle Rydback, Mamunur Rashid, Henrique Barros

**Affiliations:** 1 Department of Public Health and Sports Science, Faculty of Occupational and Health Sciences, University of Gävle, Kungsbacksvägen 47, 80176 Gävle, Sweden; 2 EPIUnit–Instituto de Saude Publica, Universidade do Porto, Rua das Taipas 135, 4050-600 Porto, Portugal; 3 Laboratório para a Investigação Integrativa e Translacional em Saúde Populacional (ITR), Rua das Taipas 135, 4050-600 Porto, Portugal; 4 Departamento de Ciências da Saúde Pública e Forenses e Educação Médica, Faculdade de Medicina, Universidade do Porto, 4200-450 Porto, Portugal; 5 Department of Business and Economic Studies, University of Gävle, Kungsbacksvägen 47, 80176 Gävle, Sweden

**Keywords:** climate change adaptation, public health, Söderhamn, Porto, Navotas

## Abstract

Climate change presents an unprecedented public health challenge as it has a great impact on population health outcomes across the global population. The key to addressing these health challenges is adaptation carried out in cities through collaboration between institutions, including public health ones. Through semi-structured interviews (*n* = 16), this study investigated experiences and perceptions of what public health aspects are considered by urban and public health planners and researchers when planning climate change adaptation in the coastal cities of Söderhamn (Sweden), Porto (Portugal) and Navotas (the Philippines). Results of the thematic analysis indicated that participating stakeholders were aware of the main climate risks threatening their cities (rising water levels and flooding, extreme temperatures, and air pollution). In addition, the interviewees talked about collaboration with other sectors, including the public health sector, in implementing climate change adaptation plans. However, the inclusion of the public health sector as a partner in the process was identified in only two cities, Navotas and Porto. Furthermore, the study found that there were few aspects pertaining to public health (water and sanitation, prevention of heat-related and water-borne diseases, and prevention of the consequences associated with heat waves in vulnerable groups such as children and elderly persons) in the latest climate change adaptation plans posted on each city’s website. Moreover, participants pointed to different difficulties: insufficient financial resources, limited intersectoral collaboration for climate change adaptation, and lack of involvement of the public health sector in the adaptation processes, especially in one of the cities in which climate change adaptation was solely the responsibility of the urban planners. Studies using larger samples of stakeholders in larger cities are needed to better understand why the public health sector is still almost absent in efforts to adapt to climate change.

## 1. Introduction

“Climate change” refers to a long-term modification of the average weather patterns that have come to define Earth’s local, regional and global climates. Changes observed in Earth’s climate since the early 20th century are primarily driven by greenhouse gas emissions [1].

Greenhouse gas emissions cause heat to be trapped by the Earth’s atmosphere, and this has been the main driving force behind global warming. The main sources of these emissions are natural systems and human activities. Natural systems that are sources include forest fires, earthquakes, oceans, permafrost, wetlands, mud volcanoes, and volcanoes [2], while human activities that contribute are predominantly related to energy production, industrial activities, and those related to forestry, land use, and land-use change [3]. The 2021 Intergovernmental Panel on Climate Change (IPCC) report indicated that there is evidence that human influence has warmed the atmosphere, land, and oceans; and that there have been widespread and very rapid changes in the atmosphere, ocean, cryosphere, and biosphere [4]. Further, it showed that human-induced climate change is already causing many kinds of weather and climate extremes in every region across the globe, resulting in recurring extreme events such as droughts, heat waves, heavy precipitation, tropical cyclones, and wildfires [4].

Climate change also presents an unprecedented public health challenge as it has a great impact on population health outcomes (physical and psychological) and on different demographic groups across all continents [5,6,7,8,9,10,11,12,13,14,15,16,17].

More than half of the world’s population currently lives in cities, and with increasing urbanization rates, more and more people are exposed to the direct impacts of climate change and the associated consequences, which affect today’s city dwellers as well as those of the future [18,19]. 

Climate change can have direct and indirect effects on population health: health can be affected directly “through extreme temperatures and shifts in precipitation patterns, causing heat waves or cold spells, storms, droughts, wildfires, and floods, and a change in the patterns of disease spread by vectors and rodents” [20,21]. Indirectly, health can be affected by changes in food sources, disruption in methods of food production, and decreased economic productivity [20,21]. It is argued that the health consequences derived from climate change are influenced by the degree to which effective adaptation measures are implemented [20].

Climate change has been associated with physical and mental health outcomes. It affects physical health through increased heat stress, injury, disease, and disruption to the food supply, and it affects community wellbeing through damage to the economic and, consequently, social fabric of communities, especially impacting socially vulnerable groups [21]. In a study carried out in Bangladesh, Ahmed and co-authors found that extreme weather events were associated with injuries in children and elderly people to a larger extent than in young and middle-aged adults [22]. In a New Hampshire, United States (US) study from 2001–2009, Neugent and colleagues reported that high temperatures (>32 degrees Celsius) were associated with increased hospital visits and all-cause injury [23]. Furthermore, Friedman and colleagues found that in Illinois, the crude annual total hospital utilization incidence rate due to heat was 23.6 per 100,000 residents compared with 23.3 due to cold injuries. However, hypothermia made up 27% of all temperature-related injuries and accounted for 94% of all temperature-related deaths during 2011–2018 [24].

Climate change increases the spread of numerous infectious diseases (especially vector- and water-borne diseases). This is mostly caused by a geographic expansion of the environmental conditions—higher temperature, precipitation, and humidity—that increase an area’s suitability for vectors [25,26,27,28,29], which are spreading to regions that used to be unsuitable [30].

In addition, more frequent droughts caused by climate change can result in food insecurity and famine, which are likewise related to worse health outcomes [31,32,33]; finally, climate change also affects air quality by (1) modifying the concentration and distribution of pollen and other allergens [34]; and (2) influencing the emission, transport, dispersion, chemical transformation, and deposition of pollutants [34]. The reduced air quality will directly affect human health and ecosystems and impact climate in a feedback loop [34].

Climate change has also been found to influence populations’ mental wellbeing [31,32,33]. This can occur as a result of traumas associated with higher frequency, intensity, and duration of climate-related disasters [7,35,36,37,38] as well as of the destruction of landscapes, which diminishes the sense of belonging and solace that people derive from their connectedness to the land [39]. For instance, in a recent study of the impact of climate change on mental health, Cianconi and colleagues found that weather changes caused psychophysical symptoms such as mood disturbances, irritability, anxiety, physical weakness, hypertension headaches, hyperalgesia and pains, and autonomic symptoms [40]. The authors reported that weather changes likely induced psychopathology (e.g., seasonal affective disorders, weather sensitivity, and meteoropathic conditions) in their study population [40,41]. In another study, carried out in 25 countries across the world, it was found that “negative climate-related emotions were positively associated with insomnia symptoms and negatively related to self-rated mental health in most countries” [38]. Reporting from California, Silveira et al. found that climate-related wildfires were associated with mental health illness sequelae (e.g., post-traumatic stress disorder (PTSD) and depression) [42].

### Public Health in Climate Change Adaptation

According to the literature, there are two strategies for addressing climate change, mitigation and adaptation. “Mitigation” refers to actions that limit the extent and rate of climate change by constraining the emissions of greenhouse gases or enhancing their sinks [43,44,45]. It entails several public health benefits, for instance, increased physical activity due to the promotion of cycling and walking and pollution reduction associated with increased non-motorized transportation. On the other hand, “adaptation” refers to initiatives and measures to reduce the vulnerability of natural and human systems to actual or expected effects of climate change. Adaptation aims to minimize the potential impacts of climate change and reduce, at the least cost, its adverse effects on health [45,46]. Furthermore, in the context of health protection, adaptation encompasses all actions that protect populations from the health impacts of climate change. For adaptation to be effective, it is necessary to understand the current and projected climate change impacts and their implications for health and then to develop and implement a range of responses to ensure an optimal level of adaptation [45,46].

Efforts to integrate health in mitigation and adaptation policies are best supported by adequate accountability and monitoring of the effectiveness of the interventions, as well as their costs. Cities may contribute to the long-term mitigation of rising temperature and air pollution through policies that reduce energy consumption in transportation, industry, and households [47], improve the built environment [48], and/or increase carbon sequestration through the preservation or creation of urban forests [49]. Examples of adaptations include improved weather forecasting, heat–health early warning systems, air quality alerts, and emergency preparedness to deal with elderly and institutionalized populations during extreme events [50,51]. Having locally integrated frameworks for climate policies that work within specific socio-ecological contexts is imperative and desirable [52]. 

With regard to public health, the Health in All Policies (HiAP) approach may be of importance in the context of climate change adaptation. This approach to public policies across sectors systematically takes into account the health and health systems implications of decisions, seeks synergies, and avoids harmful health impacts in order to improve population health and health equity. The HiAP approach is founded on health-related rights and obligations [45]. It emphasizes the consequences of public policies on health determinants and aims to improve the accountability of policy makers for health impacts at all levels of policy making [45,53]. “The goal of HiAP is to ensure that decision-makers are informed about the health, equity, and sustainability consequences of various policy options during the policy development process” [45,53]. This approach is based on the premise that good health is fundamental for a strong economy and vibrant society and that health outcomes are largely dependent on the social determinants of health, which in turn are shaped primarily by decisions outside of the health sector [53,54]. 

Incorporating health and health equity into decision-making across sectors requires intersectoral collaboration as well as changes in government organizational structures and processes to clarify, support, and advance the achievement of the priority goals of diverse stakeholders in and out of government [53,54]. For instance, solutions for complex and urgent problems such as climate change adaptation will require collaborative efforts across many sectors at local and national levels, including government agencies, businesses, community-based organizations, and non-governmental organizations (NGOs). Collaboration across sectors can also promote efficiency by identifying opportunities to share resources and reduce redundancies, thus potentially decreasing costs and improving performance and outcomes in a time of great pressure on government resources [55]. At its core, HiAP is a strategy to improve population health through intersectoral collaboration with partners who have the ability to influence the social determinants of health [45]. Thus, with regard to climate change, HiAP asks those in other sectors to recognize the impact their own work has on health, break down silos, build new partnerships to promote health, equity, and sustainability, and increase government efficiency. A HiAP approach focuses on collaboration through relationship building rather than sporadic or single-project coordination [45]. The main assumption is that collaboration requires partners to understand both the vision and the goals of the group as a whole, and the goals and objectives of each of the partners, as well as the unique perspectives, specialized expertise, concerns and constraints, and potential contributions that each partner brings [45,53]. 

Health in All Policies collaborative relationships depend not only on a shared vision and common goals but also on the practices of trust, reciprocity or generosity, and mutuality. Given that the majority of climate adaptation plans across the world’s cities are prepared by sectors other than public health, it may be important to understand how the multi-sectoral collaboration for climate change adaptation takes place in urban environments. Although there are increased threats from climate change to the urban population, few studies have attempted to investigate what health/public health aspects are being considered when drafting and implementing municipal or regional climate change adaptation plans. 

Therefore, this study aims to qualitatively investigate experiences and perceptions of what public health aspects are considered by stakeholders in the municipal climate change adaptation plans of three coastal cities located on two different continents. In addition, the study examined what public health aspects were included in the latest climate change adaptation plans published by each of the cities. 

Answers to the following questions were sought: (1) What are the perceived risks of climate change in each city? (2) Which partners are involved in climate change adaptation planning and implementation in each city? (3) What public health aspects are being included in climate change adaptation and implementation in each city as perceived by the city stakeholders? and (4) What public health-related aspects were included in the latest climate change adaptation plan report published on each city’s/municipality’s website?

## 2. Materials and Methods

### 2.1. Study Settings

The study was carried out in three cities on two continents and with different climate types and demographic characteristics: Söderhamn (Sweden), Porto (Portugal), and Navotas (the Philippines). Söderhamn city is part of Söderhamn Municipality, Gävleborg County, and covers an area of 10.53 km^2^. It has 11,761 inhabitants and a population density of 1117 km^2^ [56]. The municipality is located on the coast, and large parts of the city and its buildings are close to the water. The climate is characterized by partly cloudy summers and long, freezing, cloudy and dry winters. A rise in sea level, flooding and extreme weather events constitute the main climate change-related threats to the city [57]. 

Porto is a city in northern Portugal and the center of the Porto metropolitan area. It is 41.42 km^2^ in area, with 263,131 inhabitants and a population density of 5736 inhabitants/km^2^ [58]. The climate is temperate oceanic, with mild, rainy winters [59]. According to Monteiro and colleagues, there have been signs of seasonal disorganization, with increases in temperature, a number of extreme heat and cold events, changes in the annual distribution of precipitation, and a higher frequency of paroxysmal episodes involving both precipitation (intense precipitation or drought) and wind [60].

Navotas City is the second smallest city in metropolitan Manila in the Philippines. It has a population of 247,543 inhabitants, covers an area of 10.77 km^2^, and has a population density of 27,689 km^2^. The city is divided into 18 administrative divisions called “barangay” (districts or wards), which are independently administered by barangay captains [61]. Navotas and the rest of the Philippines have two seasons—the wet season (May to October) and the dry season (November to April). During the wet season, an average of 20 typhoons pass through the Philippines, resulting in devastating effects on low-lying cities such as Navotas [62]. 

The tropical monsoon climate is prevalent as in other parts of the Philippines [59]. Climate change threats to the city include a rise in the sea level, increased monsoon rains, typhoons, and floods [62]. The three cities have similarities (be in coastal areas), but also, they are different as two are in Europe (developed countries of the Global North) and one in Asia (developing country in the Global South). The Global Risk Index, 2021, reported that although the poorest countries in the Global South (as compared to the Global North) registered the lowest industrial pollution levels, they were more susceptible to damage produced by climate change. The report also indicated that the inequality already existing in the countries of Global South exacerbated their vulnerability to climate change impacts, thus hampering efforts for poverty reduction [63].

### 2.2. Design, Participants and Procedures 

An exploratory qualitative study was conducted. Data were collected through semi-structured interviews, which were deemed an appropriate method of data collection. The study questions sought to understand stakeholders’ motivations towards and perceptions of public health aspects of climate change adaptation. Firstly, a thematic interview guide was developed by the research team in English and subsequently translated into Swedish and Portuguese. Back translation was conducted to ensure the accuracy of the original guide. Secondly, the interview guide was pre-tested in each city for refinement of content ahead of the final data collection. The interview guide was divided into four parts: background information (the participant’s age, sex, and profession); identification of climate change-related risks; climate change adaptation planning and implementation (including planning and implementation pertaining to health and public health); and sustainable development in the context of climate change adaptation strategies.

A sample of 16 key stakeholders (*n* = 5 in Söderhamn; *n* = 6 in Porto; and *n* = 5 in Navotas), including urban public health planners and researchers, agreed to participate in the study after they were briefed on the study’s aims as well as the possibility to withdraw from the study at any stage of the interview. Further, participants were assured of their anonymity and confidentiality regarding their age and professional title. 

The interviews were carried out face to face and by telephone from February to April 2021 by F.S. in Söderhamn (Sweden), during February 2021 through the Zoom platform by M.Ry. in Navotas (the Philippines), and during March 2021 via Zoom by J.P.S. in Porto (Portugal). The interviews lasted an average of 50, 45, and 66 min, respectively. All interviews recorded on Zoom (in Navotas and Porto) were erased hours after they were transcribed into text.

### 2.3. Data Analysis 

The in-depth interviews were analyzed using the thematic analysis framework proposed by Braun and Clarke in 2006 [64]. This offers an “accessible and theoretically flexible approach to analyzing qualitative data” and is a “method for systematically identifying, organizing, and offering insight into patterns of meaning (themes) across a dataset” [63].

This framework suggests a six-phase process of analysis: (1) familiarizing with the data by transcribing and extensively reading them; (2) generating initial codes; (3) searching for themes by collating codes into potential themes; (4) reviewing the themes; (5) defining and naming the themes; and (6) producing the scientific paper. An inductive approach was used to develop key themes. The inductive approach is a bottom-up method driven by what is in the data so that what is mapped by the researcher closely reflects participants’ opinions [64].

The development of codes and themes was an iterative process conducted by researchers from the three teams in Sweden (F.S., G.M.), Portugal (J.P.S., A.I.R.), and the Philippines (M.Ry., G.M.), with the researchers discussing and revisiting the data until consensus was reached. Thereafter, the themes from the three cities were compared in order to harmonize the overall themes for the whole study. Teams compared the themes emerging from their data to harmonize the themes for the whole study. 

Document content analysis [64] was performed to analyze city adaptation plans that are publicly available in each city/on each city’s website and to identify which areas pertained to the public health arena (see Table 1).

### 2.4. Ethics

The study protocols were approved by the Swedish Ethics Committee (approval number 2020-05278), the Ethics Committee of the Public Health Institute of the University of Porto (approval number CE20175), and each city’s corresponding authority. The Navotas city authority accepted the ethical approval given by the Swedish Ethics Committee. All participants received an explanation of the purposes of the study and provided informed consent before each interview. In order to protect participants’ confidentiality, an agreement with participants was reached not to mention their age, sex, or professional category. This was in order to reduce the likelihood of being easily identified, as in the cities included in the study, very few people are involved in urban planning, health and climate change research, and/or coordination of climate change adaptation plans.

## 3. Results

This study explored the experiences and perceptions of city stakeholders regarding what public health aspects are included in climate change adaptation plans, as well as what public health aspects had been included in the latest adaptation plans published by each city’s government.

### 3.1. Experiences and Perceptions of City Stakeholders

Thematic analysis [65] of the interviews revealed four main themes regarding stakeholders’ experiences and perceptions of public health aspects related to climate change adaptation: (1) Climate change risk awareness; (2) Climate change adaptation and collaboration with other sectors, including public health; (3) Barriers to climate change adaptation; and (4) The relationship between climate change adaptation and sustainable development.

#### 3.1.1. Climate Change Risk Awareness

Stakeholders interviewed in the three cities stated they were aware of the importance of climate change as well as of the potential risks it presented for their city. This awareness ranged from how city officials understood the importance of climate change and what were the most immediate threats from climate change to their city. For instance, in Söderhamn, a participant stated that there was a great awareness of climate change, especially rising water levels:

*Söderhamn is a coastal city, so there we have the risks of water levels rising along the coast and *[threatening]* buildings along the coast, so to speak. You also have the bay and the Söderhamns River that enters* [the sea there]*. When we plan, community plan, we know about this. Or we know that we have land along the water that was previously the seabed and that is not so good to build on.*

(Participant A, Söderhamn, Sweden)

In Porto, an interviewee mentioned how people were aware of the emission of pollutant gases, even though this is a relatively recent awareness.

*Um... It’s like this: there is something that I notice a lot in my students, which is a growing awareness concerning climate change, the emission of gas pollutants, the greenhouse effect, recycling. […] I think there is a great awareness among the new generations.*

(Participant D, Porto, Portugal)

In Navotas, an interviewee also talked about how the people living there were aware of climate change threats that affected the city, especially those linked to flooding.

*Here in Navotas, we have identified some hazards such as floods, tsunamis, soil liquefaction, landslides, ground ruptures, ground shaking, and storm surges. Flooding is a perennial problem here because we are surrounded by water—Manila Bay in the west and the Malabon–Navotas River in the east.*

(Participant A, Navotas, Philippines)

#### 3.1.2. Climate Change Adaptation and Collaboration with Other Sectors, including Public Health

Participants across the cities talked about the work that is being carried out regarding climate change adaptation. Overall, they saw climate change adaptation as an ongoing process they needed to keep working on. Furthermore, in all cities, the participants talked about how they needed to collaborate with several entities, including public health, to develop climate adaptation plans. In Porto and Navotas, interviewees identified public health-related collaboration partners as part of the climate change adaptation process. 

A participant in Söderhamn mentioned how the city worked with the Baltic Sea/Water authorities responsible for the European Union (EU) projects to adapt to climate change.

*We work a lot with… with these EU projects (Baltic Sea and Water). We have got a project, I think at least among the officials, who works in Community Planning and the Technical Department, an understanding of just stormwater and water quality.*

(Participant A, Söderhamn, Sweden)

Likewise, in Porto, there was an active collaboration with other institutions to better plan for climate change adaptation. An interviewee in Porto explained how the city’s authorities collaborated with NGOs and primary health care authorities to implement some activities to address climate change:

*Many measures that we worked on related to awareness; we could not have done it without that collaboration from the primary health care provision structure (ACES) colleagues. We understood that there was a prevalence of skin cancer, excessive exposure […], and based on the climate predictions, this will probably get worse, and so we did not have any awareness action prepared. I mean, we never thought about those aspects, right? We didn’t realize the importance of sensitizing people to use skin protection, for example, right?*

(Participant C, Porto, Portugal)

In Navotas, interviewees spoke about the collaboration with many stakeholders, including social services, environmental and economic-related institutions, and the local public health authority.

*Public health is well represented when it comes to climate change planning, also in disaster risk reduction and management (DRRM) planning. Planning is done by different sectors and institutions–social, environmental, economic—these are represented by different departments. As concerns social and institutional involvement, the city health office and the Navotas city hospital are always represented. There are also plans and activities to ensure the inclusion of public health in the budgeting and allocation of funds—short and long-term.*

(Participant C, Navotas, Philippines)

#### 3.1.3. Barriers to Climate Change Adaptation

Participants in the three cities acknowledged that their city’s authorities faced an array of challenges linked to their local strategies to implement activities for climate change adaptation. These challenges included the climate change itself, as well as the need for collaboration with other institutions; financial strain; lack of fine-grained information about health effects; and low awareness of climate change adaptation. The last-mentioned challenge was due to the challenge of communicating about climate change, both with decision makers and with the general population. In Söderhamn, an interviewee expressed concerns regarding challenges faced by city authorities in terms of money, knowledge, and time when choosing what risks for climate change should have priority in the adaptation plans.

*Money is a big problem. Knowledge too can be a problem… And time. It depends on the focus you choose! What do you choose first? It is always a matter of choice.*

(Participant C, Söderhamn, Sweden)

In Porto, an interviewee reflected on the difficulties of obtaining the appropriate data indicators on the effects of climate change on health for decision making.

*We keep working with conventional indexes and indicators. [Interviewer: Uh-hm.] These are the ones that are studied, in use, and measurable. In the case of Porto, I have a big problem with the public health indicators, because I never get disaggregated information. It is possible that the reality of Eastern Porto—I don’t know for sure because I don’t have disaggregated information—is slightly different from the Foz area, or from Western Porto.*

(Participant A, Porto, Portugal)

In Navotas, lack of community awareness and difficulties in accessing national funding for climate change adaptation were some of the most important barriers faced by the city.

*Getting access to the national and international funds that are available for the climate change initiative is quite challenging for local government units. Criteria are very high and difficult to achieve for small local government units.*

(Participant A, Navotas, Philippines)

#### 3.1.4. The Relationship between Climate Change Adaptation and Sustainable Development

In the three cities, interviewees were aware of the relationship between climate change adaptation and overall sustainable development. Thoughts ranged from the potential economic costs of an unsustainable built environment to the importance of the United Nations (UN) Sustainable Development Goals (SDGs) and the holistic approach that the SDG agenda provides as a bedrock for climate change adaptation.

In Söderhamn, an interviewee pointed to the long-term economic costs related to the urban environment, which could arise because of a lack of climate change adaptation. The respondent specifically mentioned flooding, which could damage residential buildings.

*Well, there can be very substantial economic consequences if buildings or the urban environment in general are not sustainable… and if buildings are flooded and destroyed because we have not taken sufficient account of climate change, and so on. It will be expensive and … no, but it is probably also dangerous for people in different ways.*

(Participant E, Söderhamn, Sweden)

In Porto, an interviewee clearly saw a link between climate change and the SDG Agenda 2030, where SDG 13 (Climate action) is related to the other goals even at the local level.

*The 17 goals of sustainable development, where goal 13 concerns adaptation to climate change, and the national transposition of the SDGs, will be a fantastic job, a unique opportunity that we will all have, as a civilization, to make big changes. Which is the SDGs, the 17, and the effort we are making for the local SDGs, the goals of local sustainable development. So, I think we will get the world back one day.*

(Participant B, Porto, Portugal)

In Navotas city, too, interviewees were aware of the relationship between climate change adaptation and sustainable development and how together they would contribute to the quality of life for current and future generations.

*Sustainable development is the type of development that all countries are geared towards. My understanding is that it is a holistic approach in terms of development to ensure that the quality of life of the present generation, but also of future generations, should be well ensured. All the challenges that future generations will address, like poverty, food security, climate change and environmental degradation. There are a lot concerns that SDGs are trying to address. We are geared towards these types of development.*

(Participant D, Navotas, Philippines)

### 3.2. Analysis of the Latest Climate Change Adaptation Plans in Each City 

A qualitative content analysis was made of the latest climate change adaptation plans available on each city’s website to find out what aspects were mentioned pertaining to the area of public health. The climate change adaptation for Porto (2016) and Navotas (2016–2025) was developed only for the city. By contrast, in Söderhamn, the climate change adaptation plan (2020) was designed for the entire region of Gävleborg to be implemented in each city in the region. Findings showed that, compared with Söderhamn’s plans, the climate change adaptation plans of Porto and Navotas included more actions related to public health (see Table 1).

In Söderhamn, the latest adaptation plan mentioned the importance of providing sanitation and clean water in case the city experienced flooding [66]. In that city, the climate change adaptation plan priorities were set through the regional plan (which replicated the national priorities), and the main areas of concern entailed: (a) landslides and erosion that threatened communities, infrastructure, and businesses; (b) flooding, which threatens communities, infrastructure, and businesses; (c) high temperatures posing risks to human and animal health and wellbeing; (d) shortages in water supply for individuals, agriculture and industry; (e) biological and ecological impacts affecting sustainable development; (f) impact on domestic and international food production and trade; (g) and (h) increased occurrence of pests, diseases and invasive alien species affecting humans, animals, and plants. The plan also mentioned the potential consequences of climate change on children and older people but did not articulate what progress was made in the areas of concern prioritized above [66]. 

The objectives of the Porto climate adaptation plan [67] were to: (i) increase the municipality’s knowledge regarding predisposition to extreme weather events and respective adverse impacts on the safety of people and property and the key actors to mobilize in the adaptation process; (ii) reduce vulnerability to climate events and increase the adaptive capacity of the municipality; (iii) guarantee the maximum integration of the knowledge present in this document in the different territorial management instruments; (vi) orient the redesign of the city green structure, in order to guarantee an effective minimization of the effects of climate change in the municipality’s territory; (v) empower and mobilize the different actors of the civil society in the process of implementation of the strategy; and (vi) to ensure the development of all studies and actions aimed at following up and giving practical effect to this strategy [67]. However, although there is no direct follow-up to the adaptation plan, a study by Monteiro et al. found evidence of an increase in extreme heat, cold, and wind waves in the municipality of Porto [60]. Regarding public health, the city climate adaptation plan mentioned the importance of preventing the consequences of heat waves, specifically regarding improvements in thermal comfort in housing, hospitals, and childcare and elderly institutions, as well as increasing the capacity of health care institutions to respond to surges in emergency visits during and following extreme temperatures. In addition, the plan included the need to investigate and monitor vectors and create a prevention/contingency plan for vector-borne infections as well as increased population awareness of allergic diseases, sun exposure, and skin cancer, and expand green and blue spaces [67].

In Navotas, the current adaptation plan (2016–2025) named three areas related to public health: water and sanitation, water-borne diseases, and mortality due to flooding. Regarding water and sanitation, the plan pointed to the importance of providing sanitation and clean water and of preventing water-borne diseases as well as deaths due to these diseases in cases of flooding, storms, or tsunamis. Overall, in the Navotas climate adaptation plan, the main concerns were related to: (a) the intensification of rainfall, river flow, and flooding from June to November (Southwest monsoon), (b) the decrease in rainfall from December to May (Northwest monsoon) and (c) increase in mean temperature that could lead to sea level rise, increase in the sea surface temperature and stronger typhoons [68]. The plan estimated that a total of nineteen thousand inhabitants could be affected by flooding (e.g., households, businesses, etc.). Regarding adaptation activities, the plan pointed out that the city was already implementing structural and non-structural preventive (and mitigating) projects to address the concerns described above. Structural preventive measures included construction of pumping stations, construction and upgrading of drainage systems, construction of river walls, and maintenance of flood control facilities [68], and non-structural measures entailed the creation of local inter-agency/committee on waterways as well as regular de-clogging of canals [68].

## 4. Discussion

Overall, the results of the present study found that participants in the three cities were aware of the main risks posed to their city by climate change. These ranged from the heat island effect, rising water levels, and flooding to landslides, tsunamis, forest fires, and air pollution, among others. Some interviewees indicated that some segments of the general population were aware of the climate risks. Furthermore, participants mentioned that there was intersectoral collaboration, including the public health sector, in the implementation of the climate change adaptation plans, although this differed between cities. In all cities, participants identified an array of barriers to climate change adaptation, which included the complexity of climate change itself, lack of full collaboration between institutions, and financial strain. Participants also emphasized how climate change was linked to the achievement of all the UN SDGs and specifically goal 13 (“Take urgent action to combat climate change and its impact”). 

Regarding awareness of risks of climate change across cities, in Söderhamn, interviewees pointed to the heat island effect and rising water levels, and potential flooding as one of the main threats to the city. In Navotas, also a coastal city, interviewees identified storms, flooding, tsunamis, and landslides as some of the most important threats to the city; while in Porto, extreme temperatures (hot and cold temperatures and their effects in view of the poor quality of buildings, for example), rising sea levels and air pollution were the main concerns. Above all, participants saw climate change and climate adaptation as important and recognized that it was an ongoing process needed to counteract the threats to their cities; and that there was a need to improve awareness among different stakeholders (e.g., residents and politicians). It is argued that adaptation to climate change is important as it involves changes in socio-ecological systems in response to actual and expected impacts of climate change in combination with non-climatic drivers such as demographic change or economic development [69]. Moser and Ekström point out that adaptation strategies can range from short-term to longer-term activities with the aim of doing more than meeting climate change goals alone, which might or might not succeed in moderating harm and/or exploiting beneficial opportunities [69]. Moreover, Iturriza and colleagues argue that although efforts have been made to build city resilience and improve cities’ capacity to respond to, recover from and adapt to climate change, many stakeholders lack proactive behavior, which has resulted in less effective city resilience-building strategies [70]. 

With regard to intersectoral collaboration, including the public health sector, in the implementation of climate change adaptation, findings differed across the studied cities.

The interviewees in all three cities pointed out that there was an ongoing collaboration across various sectors, including public health and other health-related sectors. For instance, in Porto, there was a collaboration between city planners and NGOs, companies, and researchers. In addition, there was a strong collaboration between the climate change adaptation entities and primary health care to better understand health trends and the increase in some diseases (e.g., skin cancer). In Navotas, there also was a synchronized collaboration between all city stakeholders, including public health and health care (e.g., Navotas Hospital) and social and economic sectors in the development of climate adaptation. By contrast, in Söderhamn, the interviewees did not mention the public health sector as part of climate change adaptation efforts in the city, which left this task largely to urban planning authorities. This finding is in line with the Lancet Countdown group’s argument that, across Europe, local climate adaptation was largely siloed in specific departments, with little or no inclusion of health authorities or consideration of potential co-benefits, impacts, or unintended harms to public health [71]. Although scarce, evidence is starting to trickle down from empirical evaluation of intersectoral climate adaptation collaboration efforts across the globe. For instance, Barton et al.’s study in Santiago, Chile, regarding governance and challenges of participatory climate change adaptation found that the main challenges were linked to making a case for climate change adaptation to the involved stakeholders, including effective communication of scientific data, and clarity regarding methodologies and uncertainties; and ensuring an integrated and coordinated response rather than sectoral fragmentation [72]. 

There is now a recognition that although mitigation efforts may succeed, it is important that they are paired up with climate adaptation [4]. Hence, climate adaptation and, specifically, health adaptation and resilience are central to the reduction of health risks from climate change hazards and also minimize exposures and vulnerabilities [4,73]. It is suggested that collaboration with the public health sector is of great importance for climate adaptation in cities because although climate change per se does not create new health problems, it exacerbates and worsens known public health and clinical problems and alters the geographic patterns of disease [74].

The participants also talked about the barriers they experienced regarding the implementation of climate change adaptation. These included the complexity of climate change. As mentioned above, the participants felt that climate change, and climate change adaptation, was an ongoing process that needed to be constantly addressed and considered each day. It is argued that the complexity of climate change arises from the uncertainty of climate projections that can make it difficult to deal with uncertainties of future demographic, socioeconomic, and technological conditions that might change the exposure, sensitivity, and adaptive capacity of the population [74]. 

In addition, the interviewees expressed concerns about difficulties in achieving collaboration across different sectors. This was the case, especially in Söderhamn, where there was almost no mention of collaboration with sectors such as the public health sector. Here, the majority of climate adaptation planning was performed solely by the urban planning entities. Some authors have pointed to the complexity of navigating the formal and informal rules and organizational arrangements governing collaboration [75,76,77,78,79]; also, they have addressed issues related to individual cognition (e.g., individual knowledge of climate change is necessary, but individual knowledge alone is insufficient for adaptation) [78,80] and social capital (this enables residents to coordinate community action to achieve shared goals) [81].

In Söderhamn and Navotas, participants identified financial strain as one of the main barriers to climate change adaptation. They said that funding usually went to bigger cities as they were more likely to meet the established criteria compared with small cities (such as Navotas). Moreover, interviewees in Navotas described the difficulties faced by the city in convincing residents in informal settlements of the importance of climate change adaptation efforts. According to Agrawala and Fankhauser, managing both current and future effects of climate change can, and will, be costly [82]. Addressing the financial concerns linked to climate change adaptation mentioned by interviewees is even more important for low- and middle-income countries, which bear most of the burden of climate change risks [78]. Furthermore, participants in Navotas talked about the difficulties of intersectoral collaboration, despite the fact that climate change effects are upon us. 

In all three cities, interviewees were unanimous in recognizing a link between climate change adaptation and the overall SDG agenda. In Söderhamn and Navotas, interviewees pointed to the important link between climate change and the economic and environmental aspects of sustainable development and described how making development sustainable would lead to healthier and sustainable societies. In addition, interviewees in Navotas emphasized that climate change adaptation needs to go hand in hand with a reduction of poverty and environmental degradation and improved food security for today’s generation and future generations. It has been argued that impacts of climate change might make some development targets harder to achieve (e.g., impacts of climate change on agricultural production can set back efforts to reduce poverty and hunger) [83,84]. Furthermore, actions to adapt to climate change can also directly interact with other development goals, for instance, involving both positive synergies and negative trade-offs [85,86,87,88,89,90,91]. Moreover, evidence from analysis of data from diverse economic and social, and country contexts has shown that outcomes of climate action can have differential impacts on vulnerable social groups, including extreme cases where national climate adaptation programs have resulted in the violent displacement of poor communities [92].

With regard to the analysis of the latest climate change adaptation plans available on the website of the three cities, we found that there were few public health adaptive initiatives. However, there was mention of water and sanitation and of the need to prevent water-borne diseases and provide protection against heat waves and certain diseases (e.g., allergies and skin cancer). 

The lack of inclusion of public health aspects in urban settings has been reported elsewhere [93,94,95]. For instance, a recent comparative review, which aimed to characterize the public health role in the adaptation plans of 22 large cities pre-identified as highly health-adaptive, examined five health-associated adaptation activities as “promising practices” based on evidence synthesized from evaluation research and practical experience [95]. The five areas included: hazard and vulnerability mapping; extreme weather preparedness and response; extreme heat plans (including heat early warning); non-heat early warning (e.g., flooding or vector-borne diseases); and climate–health monitoring and outcome surveillance. Results of that study showed that 90% of the cities’ adaptation plans reported actions in at least three of the five activity areas but that only 73% of the health-focused plans reported involvement of a public health agency (although the share was higher for cities in low- and middle-income countries) [95]. Furthermore, a study that assessed the state of health adaptation in 401 urban areas globally found that only 10% of the sample reported any public-related initiatives, and these initiatives entailed addressing risks posed by weather events and aspects related to changes in management or behavior rather than capacity building, research, or long-term investments in infrastructure [93]. The authors also found important gaps in the health adaptation plans, which were related to limited reporting of what in reality was performed at the municipal level (especially in countries of the Global South); as well as an absence of information-based adaptation initiatives, limited focus on initiatives addressing disease risks, and absence of monitoring, reporting and evaluation [93]. 

### Strengths and Limitations

This study has important strengths: (1) it comparatively assessed the experiences and perceptions of key stakeholders involved in climate change adaptation implementation and research in three cities, two located in Europe and one in Asia; and (2) it investigated which public health aspects were both perceived to be part of and written in the climate change adaptation plans of the studied cities.

However, the study has limitations. It is based on a purposively based sample of stakeholders with a familiarity with the subject under study in relatively small cities. Consequently, results cannot be generalized to other (e.g., larger) cities located in the same region or to other cities in the three countries or elsewhere in the world. Nevertheless, we strongly believe that this exploratory study provides an insight into the experiences and perceptions of key stakeholders on how public health aspects are being included and/or excluded in climate change adaptation plans of different cities.

## 5. Conclusions

In this study, participants in the three cities were aware of the main climate risks to their own cities, which included rising water levels, extreme temperatures, flooding, and air pollution. Furthermore, they perceived that there was some collaboration with other sectors, including public health, in the implementation of climate change adaptation plans for their cities. However, the inclusion of the public health sector as a partner in the process was identified only in Navotas and Porto. Furthermore, the study found that there were few aspects pertaining to public health in the latest climate change adaptation plans for each of the three cities. These included water and sanitation, prevention of heat-related and water-borne diseases, and prevention of the consequences associated with heat waves in vulnerable groups such as children and the elderly. It is noteworthy that the narratives from the interviewed stakeholders and the analysis of the written climate plans pointed to difficulties in intersectoral collaboration for climate change adaptation. Lack of funding for climate adaptation activities was a recurrent issue raised by participants across the different cities. The study also found that the public health sector was not fully included in the adaptation processes, especially in one of the cities in which climate change adaptation was solely the responsibility of the urban planners. Hence, there is a need for further (qualitative and quantitative) studies using larger samples of stakeholders in bigger cities to better understand why the public health sector is still almost absent in efforts to adapt to climate change.

## Figures and Tables

**Table 1:** Public health aspects of climate change adaptation plans published on the websites of the cities of Söderhamn (Sweden), Porto (Portugal) and Navotas (Philippines).

City	Climate Adaptation Plan Title	Publication Year of Current Adaptation Plan	Year of Initial Publication of the Climate Adaptation Plan	Coverage of the Plan: City/Municipality/ Region	Public Health-Relevant Content	Description of the Health-Relevant Content
Söderhamn	Handlingsplan för Länsstyrelsen Gävleborgs arbete med klimatanpassning (Regional climate adaptation plan for the Gävleborg Region)	2020	2014	Region (there is no specific city/municipal strategy. The climate adaptation plan is regional and implementation is mandatory for all cities in the region.)	Water and sanitation	Provision of water and sanitation to the population if the city/municipal area is flooded.
Porto	Estratégia municipal de adaptação às alterações climáticas (Municipal strategy for adaptation to climate change)	2016	2016	City/municipality	Heat waves and vector-borne infections	Improvements in thermal comfort in housing, hospitals, and child care and elderly institutions; increased capacity of health care institutions to respond to surges in emergency visits during/following extreme temperatures; monitoring of vectors and creation of a prevention/contingency plan for vector-borne infections; increased population awareness of allergic diseases, sun exposure and skin cancer; expansion of green and blue spaces.
Navotas	Disaster risk reduction and climate change adaptation profile	2016–2025	2016	City/municipality	Water and sanitation	Provision of water and sanitation to the population if the city is flooded.
					Water-borne diseases	Prevention of morbidity and mortality due to water-borne diseases.

## Data Availability

Study approval and participant consent forms stated that study response information would not be shared outside of the research team except in aggregate form. This was deemed prudent given the sensitive nature of the questions asked. Therefore, it will not be possible to make participant data available to others at this time. Furthermore, two other cities are currently collecting data using the same interview guide to conduct country-based studies, therefore the interview guide cannot be published as supplement.
